# Impact of augmented reality assisted navigation on early outcomes in reverse shoulder arthroplasty: a retrospective cohort study

**DOI:** 10.1186/s12891-026-10031-8

**Published:** 2026-06-08

**Authors:** Agahan Hayta, Cagman Seker, Soraya Bahlawane, David Alexander Back, Alp Paksoy, Yamac Akgun, Rony Orijit Dey Hazra, Doruk Akgun

**Affiliations:** 1 Center for Musculoskeletal Surgery, Charité Universitätsmedizin Berlin, Augustenburger Pl. 1, Berlin, 13353 Germany; 2 Department for Pathology and Laboratory Medicine, University of Miami Miller School of Medicine, Miami, Florida United States of America

**Keywords:** Reverse shoulder arthroplasty, Intraoperative navigation, Augmented reality

## Abstract

**Aims:**

Augmented reality–assisted intraoperative navigation (ARIN) has recently emerged as a novel technology intended to improve intraoperative accuracy and workflow in reverse shoulder arthroplasty (RSA). This study evaluated the impact of ARIN on early complications and revisions, glenoid baseplate inclination and fixation, fluoroscopy time, and surgical duration.

**Methods:**

Two hundred three primary RSAs were retrospectively analyzed: 72 performed with ARIN and 131 freehand. The primary outcome was 90-day complications and revisions. Secondary outcomes were baseplate inclination (RSA angle on true AP radiographs), number and length of peripheral screws, fluoroscopy time, and surgical duration.

**Results:**

No statistically significant differences were observed between the ARIN and freehand groups regarding complications (3% vs. 9.4%, *p* = .139), revisions (3% vs. 8.5%, *p* = .216), or dislocations (1.5% vs. 3.4%, *p* = .655). Baseplate inclination was significantly closer to neutral in the ARIN group (4.6° ± 7° vs. 11.6° ± 8.1°, *p* < .001), and superior inclination > 10° was significantly less frequent (18.8% vs. 55.6%, *p* < .001). Significantly fewer screws were used for baseplate fixation (2.1 ± 0.4 vs. 3.2 ± 0.9, *p* < .001), which were significantly longer (33.7 ± 6.3 mm vs. 25.6 ± 7.8 mm, *p* < .001). Fluoroscopy time (19.7 ± 15.4 s vs. 45.9 ± 37.1 s, *p* < .001) and operative duration (104.1 ± 27.3 min vs. 113.4 ± 35.3 min, *p* = .038) were also significantly shorter with ARIN.

**Conclusion:**

ARIN in RSA was associated with improvements in glenoid baseplate inclination, screw placement, intraoperative fluoroscopy time, and surgical duration, while no statistically significant differences in early complication, dislocation, or revision rates were observed compared with the conventional freehand technique. Larger studies with greater statistical power and longer follow-up are warranted to determine potential long-term clinical benefits.

**Evidence level and study design:**

Level of evidence III; retrospective comparative study.

## Introduction

Accurate positioning and secure fixation of the glenoid component in reverse shoulder arthroplasty (RSA) are essential to maximize range of motion and minimize complications [1–8]. Positioning errors have been reported frequently using standard instrumentation, even in cases performed by experienced surgeons [8, 9]. To address this challenge, solutions such as patient-specific instrumentation (PSI) and intraoperative navigation have been introduced [10].

Recent studies have demonstrated significant benefits of intraoperative navigation in RSA. In terms of glenoid positioning, intraoperative navigation has been shown to reduce deviations from the preoperative plan [11], reduce inclination and version error [12, 13], promote the use of augmented glenoid components [14], and narrow the precision gap between less experienced and more experienced surgeons [12]. Improved accuracy in glenoid component placement may contribute to a reduction in complications, particularly instability and aseptic glenoid loosening [15, 16]. Furthermore, intraoperative navigation facilitates optimal baseplate fixation by enabling the use of longer screws with ideal trajectories, thereby reducing the number of screws required while increasing screw purchase and promoting more secure, less invasive fixation [17].

More recently, augmented reality (AR) has been implemented as an additional visualization modality within computer-navigated systems [18]. While the elimination of physical guides and real-time intraoperative feedback represent general advantages of navigation over PSI, augmented reality–assisted intraoperative navigation (ARIN) further enhances these capabilities by providing intuitive head-up visualization with virtual, real-time overlay of instrument position and planned component placement directly within the surgeon’s field of view [19]. Nevertheless, ARIN, like conventional computer navigation, is associated with certain limitations, including increased costs, limited availability, technical difficulties during system operation, and a potential increase in surgical duration [16, 19, 20].

To date, while the benefits of conventional intraoperative navigation have been increasingly reported [13, 15, 17, 19, 21], clinical data specifically addressing ARIN in RSA remain scarce [22–24]. Therefore, this study seeks to provide novel clinical evidence on the technical aspects, intraoperative performance, radiographic outcomes, and safety in the early postoperative period associated with ARIN.

In this retrospective comparative study, we hypothesized that the use of ARIN would reduce short-term (90-day) complication and revision rates. Furthermore, we hypothesized that ARIN would reduce superior inclination of the glenoid baseplate, decrease the number of screws required for baseplate fixation, increase screw length, reduce intraoperative fluoroscopy time, and achieve these benefits without prolonging surgical duration.

## Methods

### Ethical approval and patient consent

This study was conducted in accordance with the principles of the World Medical Association Declaration of Helsinki. Ethical approval was obtained from the institutional ethics committee of Charité – Universitätsmedizin Berlin (approval number: EA2/037/25). Written informed consent was obtained from all patients prior to inclusion in the study.

### Patient selection

A retrospective review was conducted at our institution to identify all patients who underwent primary RSA between January 2022 and January 2025. A total of 203 cases were identified and included in the study. For the analysis of complication and revision rates, only patients with a minimum follow-up of 90 days were considered. Twenty patients were lost to follow-up (6 ARIN, 14 freehand), leaving 183 cases available for these analyses.

Of the 203 procedures, 72 (35.5%) were performed with ARIN (ARIN group), and 131 (64.5%) were performed using the conventional freehand technique (freehand group). All ARIN procedures utilized the Medacta NextAR Shoulder System (Medacta International SA, Castel San Pietro, Switzerland). The freehand group included implants from multiple manufacturers: Medacta Shoulder System (*n* = 64, 48.9%) (Medacta International SA, Castel San Pietro, Switzerland), Aequalis Ascend Flex (*n* = 46, 35.1%) and Aequalis Reversed II (*n* = 13, 9.9%) (Stryker Co., Portage, MI, USA), and Univers Revers (*n* = 8, 6.1%) (Arthrex Inc., Naples, FL, USA).

### Data collection

Patient data were extracted from the institutional electronic medical records. Collected demographic variables included date of birth, age at the time of surgery, and sex. Preoperative clinical variables comprised diagnosis, affected side, history of previous surgery on the ipsilateral shoulder, and classification as primary or revision procedure. Preoperative true-anteroposterior (AP) view radiographs were available for 54 of 72 patients in the ARIN group and 102 of 131 patients in the freehand group. In total, 18 radiographs in the ARIN group and 29 radiographs in the freehand group were excluded because they were not true-AP views. On the included images, the preoperative RSA angle, as described by Boileau et al. [7], was measured.

Intraoperative data included implant type and components, the number and length of peripheral screws used for baseplate fixation, intraoperative fluoroscopy time and surgical duration.

Postoperative adverse events (overall complications, dislocations, revisions) were assessed based on existing follow-up data and included complications and revision surgeries occurring within the first 90 days postoperatively.

Postoperative radiographs in true-AP view were available for 69 of 72 patients in the ARIN group and 124 of 131 patients in the freehand group. Postoperative RSA angle was measured on included images as described by Boileau et al. [7]. All measurements were performed by a fellowship-trained shoulder and elbow surgeon (R.D.H.) using imaging platform Visage version 7.1 (Visage Imaging GmbH, Berlin, Germany).

### Statistical analysis

Statistical analysis was performed using IBM SPSS Statistics version 29.0.1.1 (IBM Corp., Armonk, NY, USA). Descriptive statistics are reported as means with standard deviations (SD) for continuous variables and absolute numbers with percentages for categorical variables. For postoperative adverse events, only patients with at least 90 days of follow-up were included. Because complication and revision events occurred at low frequencies, categorical outcomes were compared using Fisher’s exact test, which is appropriate for small sample sizes and sparse event distributions. Absolute risk reduction (ARR) with 95% confidence intervals (CIs) was calculated according to Newcombe’s method. Continuous secondary outcomes (e.g., number of screws, screw length, baseplate inclination, surgical and fluoroscopy duration) were assessed for normality using the Shapiro–Wilk test and compared between groups using independent-samples t-tests. Mean screw length was analyzed at the patient level by calculating one mean screw length value per patient to avoid violation of statistical independence assumptions. Analyses of screw length frequencies (e.g., screws > 30 mm and < 20 mm) were performed descriptively at the individual screw level. Given the retrospective exploratory design and heterogeneity between groups, no multivariable adjustment or propensity score matching was performed. A two-sided *p*-value < 0.05 was considered statistically significant.

## Results

The study cohort consisted of 203 patients, including 132 females (65%) and 71 males (35%), with a mean age of 71.7 ± 9.2 years at the time of surgery. The right shoulder was operated on in 116 cases, and the left in 87 cases. The indications for RSA in both groups are summarized in Table 1.

**Table 1:** List of indications in freehand and ARIN groups

ARIN (*n* = 72)	Freehand (*n* = 131)
Primary OA (*n* = 31, 43.1%)	CTA (*n* = 38, 29%)
CTA (*n* = 23, 31.9%)	Acute fracture (*n* = 33, 25.2%)
Fracture sequelae (*n* = 8, 11.1%)	Primary OA (*n* = 32, 24.4%)
Irreparable rotator cuff rupture (*n* = 3, 4.2%)	Fracture sequelae (*n* = 13, 9.9%)
Chronic instability (*n* = 3, 4.2%)	Chronic instability (*n* = 7, 5.3%)
AVN (*n* = 1, 1.4%)	Irreparable rotator cuff rupture (*n* = 3, 2.3%)
Autoimmune arthritis (*n* = 1, 1.4%)	Autoimmune arthritis (*n* = 2, 1.5%)
Synovial chondromatosis (*n* = 1, 1.4%)	Synovial chondromatosis (*n* = 1, 0.8%)
Acute fracture (*n* = 1, 1.4%)	Septic arthritis (*n* = 1, 0.8%)
	AVN (*n* = 1, 0.8%)

*CTA* cuff tear arthropathy, *OA* osteoarthritis, *AVN* avascular necrosis

In the ARIN group, two complications (3%) were observed within the first 90 days postoperatively: one dislocation and one hematoma. In the freehand group, 11 complications (9.4%) occurred: four dislocations, two infections, two hematomas, two periprosthetic glenoid fractures, and one intraoperative ulnar nerve injury. The difference in overall complication rates between groups was not statistically significant (3% vs. 9.4%, *p* = .139). Similarly, no significant difference was found in dislocations specifically (1.5% vs. 3.4%, *p* = .655). Revision rates also did not differ significantly between the ARIN and freehand groups (3% vs. 8.5%, *p* = .216). Revision procedures performed in each group are shown in Table 2.

**Table 2:** Revision procedures in the freehand group and the ARIN group

ARIN group (*n* = 66)	Freehand group (*n* = 117)
Open reduction with component exchange (*n* = 1, 1.5%)	Open reduction with component exchange (*n* = 4, 3.4%)
Hematoma evacuation (*n* = 1, 1.5%)	Septic revision (*n* = 2, 1.7%)
	Hematoma evacuation (*n* = 2, 1.7%)
	Secondary bony glenoid augmentation with component exchange (*n* = 2, 1.7%)
Total (*n* = 2, 3%)	Total (*n* = 10, 8.5%)

Postoperative RSA angle was significantly lower in the ARIN group than in the freehand group (4.6° ± 7° vs. 11.6° ± 8.1°, *p* < .001). Baseplates with a superior inclination > 10° were more frequently observed in the freehand group (55.6% vs. 18.8%, *p* < .001).

The ARIN group had significantly fewer peripheral screws for baseplate fixation compared to the freehand group (2.1 ± 0.4 vs. 3.2 ± 0.9, *p* < .001), with a significantly higher mean screw length (33.7 ± 6.3 mm vs. 25.6 ± 7.8 mm, *p* < .001). In the ARIN group, 88.2% of all screws were longer than 30 mm, whereas 44.5% of screws in the freehand group exceeded 30 mm. Conversely, screws shorter than 20 mm accounted for 3.3% of all screws in the ARIN group and 30.8% in the freehand group.

The ARIN group demonstrated a significantly shorter intraoperative fluoroscopy time (19.7 ± 15.4 vs. 45.9 ± 37.1 s, *p* < .001), as well as surgical duration (104.1 ± 27.3 vs. 113.4 ± 35.3 min, *p* = .038) compared to the freehand group.

Detailed comparisons of patient demographics, postoperative adverse events, radiographic measurements, baseplate inclination and fixation, as well as intraoperative fluoroscopy time and surgical duration between the freehand and ARIN groups are presented in Table 3.

**Table 3:** Comparison of baseline demographics, radiographic, intraoperative, and clinical outcomes between freehand and ARIN groups

	ARIN	Freehand	ARR (95% CI)	OR (95% CI)	*P*-value
Patient demographics					
Age at surgery, y	71.08 ± 9.8	72.8 ± 8.9	n/a	n/a	0.210
Female sex, % (*n*)	54.2% (39/72)	71% (93/131)	n/a	n/a	**0.016**
Postoperative adverse events*					
Complications	3% (2/66)	9.4% (11/117)	6.4% (-2.0, 13.4)	0.30 (0.06–1.40)	0.139
Dislocations	1.5% (1/66)	3.4% (4/117)	1.9% (-5.0, 7.1)	0.43 (0.05–3.97)	0.655
Revisions	3% (2/66)	8.5% (10/117)	5.5% (-2.8, 12.4)	0.33 (0.07–1.57)	0.216
Radiographic outcomes**					
Pre-op RSA Angle, degrees	24.9 ± 9.6	25.9 ± 9.7	n/a	n/a	0.546
Post-op RSA Angle, degrees	4.6 ± 7	11.6 ± 8.1	n/a	n/a	**< 0.001**
Baseplate with superior inclination > 10°	18.8% (13/69)	55.6% (69/124)	36.8% (22.9, 48.1)	0.19 (0.09–0.39)	**< 0.001**
Baseplate fixation					
Screw number	2.1 ± 0.4	3.2 ± 0.9	n/a	n/a	**< 0.001**
Screw length, mm	33.7 ± 6.3	25.6 ± 7.8	n/a	n/a	**< 0.001**
Screws > 30 mm, % (*n*)	88.2% (135/153)	44.5% (182/409)	n/a	n/a	**n/a**
Screws < 20 mm, % (*n*)	3.3% (5/153)	30.8% (126/409)	n/a	n/a	**n/a**
Time parameters					
Fluoroscopy duration, s	19.7 ± 15.4	45.9 ± 37.1	n/a	n/a	**< 0.001**
Surgery duration, m	104.1 ± 27.3	113.4 ± 35.4	n/a	n/a	**0.038**

Values in the first two columns are shown as mean ± standard deviation or % (event count/*n*), as appropriate

Bold values indicate statistical significance (*p* < .05)

*ARR* absolute risk reduction, (Freehand – ARIN); *CI* confidence interval, *OR* odds ratio, *y* years, *m* minutes, *s* seconds

*Only patients with ≥ 90 days of follow-up were included for postoperative adverse events

**Only patients with true AP view radiographs were included for pre- and postoperative RSA Angle

## Discussion

In this study, we aimed to evaluate the impact of ARIN on early complications, baseplate positioning, fixation parameters, and intraoperative efficiency in reverse shoulder arthroplasty. While ARIN did not result in statistically significant differences in early (first 90 days) complication, dislocation, or revision rates compared with the conventional freehand technique, it was associated with significantly closer-to-neutral baseplate inclination, fewer peripheral screws combined with longer screw length, as well as reduced intraoperative fluoroscopy time and surgical duration.

To date, only a limited number of clinical studies have examined the impact of intraoperative navigation on complication and revision rates in RSA, and none have incorporated augmented reality. Although postoperative adverse event rates did not differ significantly between groups, the present study provides the first clinical data on augmented reality–assisted navigation in RSA, demonstrating comparable complication and revision rates to conventional techniques within the first 90 postoperative days. In a large retrospective series, Youderian et al. reported a postoperative complication rate of 2.3% in 533 navigated RSAs, compared to 3.9% in a freehand control group of 1,066 cases at a minimum 2-year follow-up [21]. Notably, the dislocation rate was significantly lower in the navigated cohort, dropping from 0.7% to 0% [21]. Similarly, Holzgrefe et al. observed reduced complication and revision rates in a matched cohort study with a minimum 2-year follow-up, with complication rates of 5.3% in the freehand group versus 1.8% in the navigated group, and revision rates of 3.5% versus 0.9%, respectively [15].

The most prominent difference observed was the significantly closer-to-neutral glenoid baseplate inclination achieved with ARIN. Recent studies have reported that ARIN significantly improves glenoid baseplate positioning compared to preoperative planning alone [9–11, 13, 14, 21, 25–29], particularly in inclination [12, 24]. The current findings are in line with this growing body of literature and emphasize the value of intraoperative navigation in improving surgical precision during glenoid baseplate implantation [13, 26]. Clinically, this is highly relevant, as superior inclination of the glenoid component has been associated with increased risk of postoperative instability, aseptic loosening, and scapular notching [2, 7, 18, 30, 31].

The ARIN group also required significantly fewer screws for baseplate fixation, and the screws used were significantly longer. Intraoperative peripheral screw navigation with ARIN may facilitate optimized screw trajectory and placement, potentially allowing improved screw purchase while preserving glenoid bone stock. A systematic review by Velasquez Garcia et al. found a pooled mean increase in screw purchase length of 5.8 mm with navigation, along with a reduction of 0.55 screws per case and a threefold increase in cases fixed with only two screws [18]. Biomechanical and comparative studies suggest that longer screws may improve baseplate fixation, and navigation may facilitate longer screw purchase with fewer screws through optimized screw trajectory [13, 32]. However, whether these technical advantages consistently translate into superior clinical outcomes or improved implant survivorship remains unclear and requires further clinical investigation.

Importantly, intraoperative fluoroscopy time was significantly reduced in the ARIN group compared to the freehand group, likely reflecting greater confidence in baseplate positioning and fixation enabled by the enhanced visual guidance of the augmented interface. Improved intraoperative visualization has previously been associated with enhanced technical execution and operative workflow in shoulder surgery [33]. To our knowledge, this is the first clinical study to report the effect of intraoperative navigation on fluoroscopy time or radiation exposure in RSA. Existing literature indicates that orthopedic surgeons are exposed to substantial cumulative radiation during intraoperative fluoroscopy [34], with some reports even suggesting an elevated risk of radiation-associated malignancies [35, 36]. Therefore, the observed reduction in fluoroscopy time may translate into meaningful decreases in radiation exposure for both surgeons and patients, potentially reducing the risk of radiation-related occupational health hazards. Given the cumulative nature of occupational radiation exposure over a surgeon’s career, this reduction in fluoroscopy utilization may represent a clinically relevant benefit of navigated and augmented reality–assisted workflows.

In the present study, the ARIN group demonstrated significantly reduced surgical duration compared to the freehand group. Concerns about increased surgical duration remain one of the primary barriers to the broader adoption of intraoperative navigation in RSA, as prolonged procedures are known to be associated with higher complication rates in total joint arthroplasty [37]. Some recent studies reported significantly longer surgical durations in navigated RSA [20, 21]. In contrast, Wang et al. demonstrated a steep learning curve for navigation, with a significant reduction in surgical duration after just eight navigated cases [29]. According to the results of our study, once familiarity with the ARIN system is established, the time spent on system initiation and registration may be offset by the time saved during baseplate positioning and fixation.

### Strengths, limitations, and future research directions

This study provides, to our knowledge, one of the first analyses specifically evaluating ARIN in reverse shoulder arthroplasty. In addition to assessing early postoperative adverse events, it includes detailed radiographic parameters and evaluates the impact of ARIN on intraoperative technical aspects such as surgical duration, fluoroscopy use, baseplate positioning, and fixation in a relatively large single-center cohort.

This study has several limitations. First, its retrospective design carries inherent risks of selection bias and limits the ability to control for potential confounding variables. Second, the ARIN group presented with a more limited and homogeneous indication profile. Third, while the ARIN group utilized a standardized implant system, the freehand group included multiple systems from different manufacturers, potentially introducing variability in instrumentation, implant geometry, baseplate design and surgical duration. In addition, the study may be underpowered to detect statistically significant differences in relatively infrequent adverse events such as complications, dislocations, and revisions. Moreover, the absence of clinical outcome measures—such as postoperative range of motion and patient-reported outcomes—limits the ability to correlate precision with functional or implant-related outcomes. Finally, follow-up was restricted to the early postoperative period (< 90 days), which represents a major limitation, precluding conclusions regarding mid- to long-term complications, implant survivorship and sustained functional benefit.

Future studies with prospective designs, larger sample sizes, and longer follow-up are warranted to further evaluate the clinical impact of ARIN in reverse shoulder arthroplasty. In particular, the inclusion of patient-reported outcome measures, functional assessments, and long-term implant survival data will be essential to determine whether the observed improvements in radiographic and intraoperative parameters translate into meaningful clinical benefits.

### Clinical implications

The findings of this study suggest that the use of ARIN in reverse shoulder arthroplasty may improve the accuracy of glenoid baseplate positioning and fixation while reducing intraoperative fluoroscopy use and surgical duration. Although no significant differences in early complication or revision rates were observed, the improved alignment and intraoperative efficiency may be clinically relevant, as accurate baseplate positioning and secure fixation are considered key factors for implant longevity and functional outcomes. In addition, reduced surgical duration may contribute to lower perioperative risk and improved operating room efficiency, while decreased fluoroscopy time may reduce radiation exposure for both patients and the surgical team.

## Conclusions

ARIN in RSA was associated with improvements in glenoid baseplate inclination, screw placement, intraoperative fluoroscopy time, and surgical duration, while no statistically significant differences in early complication, dislocation, or revision rates were observed compared with the conventional freehand technique. Larger studies with greater statistical power and longer follow-up are warranted to determine potential long-term clinical benefits.

## Data Availability

The datasets analyzed during the current study are available from the corresponding author on request.
